# Remarks on the Apothecaries' Bill

**Published:** 1813-12

**Authors:** 


*To the Editor of the Medical and Physical Journal.*

SIR,

A COPY of the following letter has been forwarded by post to the Chairman of the Committee of Apothecaries; but as it contains some facts which I conceive are not generally known, I should feel obliged by your giving it a place in the Medical and Physical Journal.

*Clifton, Bristol,* I remain your obedient Servant,
*Oct.* 24, 1813. C. W. S.

*To George Man Burrows, Esq.*

SIR,

THE great scheme of medical reform, at the head of which you are placed, seems to have excited that degree of interest interest among medical men which its importance so justly demands; and, notwithstanding the diversity of opinions that exist among them, as to the best mode of carrying it into effect, all who are not otherwise biassed by interest are agreed that the measure is absolutely necessary.

If a too great attention to private interest were the principal cause of the failure of the original bill in parliament, the one which is now to be proposed cannot meet with the same fate; because those obnoxious articles which it contained, that would have affected so deeply the interest of one set of men, and was alleged by another to be an encroachment on their privileges, are left out.

The avowed intention of this letter is not to dissent from the substance of the new bill, the adequacy of which, for the attainment of what it is ultimately intended, I very readily acknowledge; but I wish to submit to your consideration an amendment to one of its paragraphs, and to propose another, which may have the combined effect of making the medical profession still more respectable, and of bringing over to your interest those who now appear to be decidedly hostile to it.

The great mass of medical men in this country are those who practise in the three branches of the profession, compared with whom the apothecaries, in point of number, bear a very small proportion, and even these are men of respectability, by no means inferior to the surgeon-apothecary either in education or in professional knowledge; so that I believe I may assert, without fear of contradiction, that ignorance has had no share in the limitation of their practice to medicine alone; in short, as there is no sort of difference between the visiting-apothecary and the surgeon-apothecary, (the druggists of the present day being what the apothecaries formerly were,) I think the bill might with a great deal of propriety be termed that of the general practitioner in medicine, surgery, and midwifery, in which the apothecary would be considered as included. Had this been originally adopted, the pen of scurrility, guided as it has been by the worst of passions, jealousy and avarice, could not have been called into action, because it would have wanted a subject on which to ground its sarcastic aspersions; the character of the general practitioner was always respectable, whilst the regular apothecary of the present day retains little else than the name of his predecessor.

The bill which you have proposed will, if it succeed, erect another medical tribunal in this country, which, notwithstanding its inferiority, will be derogatory to the interest, if not to the privileges, of the College of Surgeons; this accounts in the most satisfactory manner for the hostility evinced by that that corporate body to the proposed bill, rightly judging that the generality of youths are not so emulous of honor, as they are of carrying into the country a certificate of their qualifications from an accredited body, and which no doubt they will attain in the easiest and least expensive manner. On this account it is that I would recommend to your serious consideration an amendment to the paragraph which I allude to, namely, instead of the new medical board being allowed to grant certificates for the addition of surgery to medicine, I would have it enacted, that all who intend to practise as surgeons, surgeon-apothecaries, or as apothecaries only, shall previously obtain a diploma from the Royal College of Surgeons; and then, *and not till then*, they may undergo another examination as to their knowledge in medicine, midwifery, &c.: in short, the general practitioner should undergo the same examinations (with the addition of midwifery) as the person who is qualified to act as full surgeon in the navy.

It may be asked, why should the visiting apothecary be obliged to undergo the same examination, with respect to surgery, as one who intends to practise exclusively in that branch? It is not for me to tell you, Sir, how frequently constitutional diseases arise from local causes, and how impossible it would be, in a case of this kind, for a practitioner, educated only in medicine, to discriminate between the cause and the effect, on which unquestionably the life of his patient would depend.

I am aware that the examinations, instituted by the College of Surgeons, are not well calculated to discriminate between the ignorant and the well-informed, the industrious and the indolent. It is far from my intention to pass any censure on the respectable body which constitutes the court of examiners; acting on the established rules of the college, they no doubt do every thing in their power to detect imposture; but the system on which the examinations are grounded is, in my opinion, radically defective. A great number of young men who call themselves medical or surgical students, care not how they get through their studies, so long as they can ultimately gain a sufficient degree of knowledge to pass their examination; but how do they acquire this? not by dissection, for it is unnecessary; but from books, and their teachers, who further their views, and confirm them in their indolence, by giving them concise answers to particular questions; they get off by rote the origin and course of the nerves of the brain; the names of vessels that arise from the principal arteries; the contents of the mediastina, Glisson's capsule, and of their different cavities; cavities; the origin and insertion of the abdominal muscles, and those of the extremities, thorax, &c.; in short, book anatomy, an attention to arrangement, and a certain degree of facility in answering questions, together with a slovenly dissection of an extremity or two merely to get a dissector's certificate, are qualifications that will allow their possessor to pass an examination in anatomy at the College of Surgeons, with perhaps more eclat than one who, disdaining such a mode of acquiring knowledge, has learnt it (where it only can be learnt) in the dissecting-room.

The evil which I have pointed out, (and I consider it to be a very great one) can only be remedied by instituting a course of examinations in anatomy over the dead subject, making one examinant point out the relative situation of parts, another to cut down on some particular artery, another to dissect the brain, &c. &c. By these means the court of examiners would be enabled to discriminate between one who has gained a practical knowledge of anatomy, and one who has acquired it in the manner which I have pointed out; and thus they would be able to reward merit, and to stigmatise those who have improperly employed their time.

There still remains another evil to be pointed out, although the remedy for it is not so apparent as for that which I have endeavored to show: it is the disgraceful practice, so notorious among all the classes in London, of carrying forged certificates with them, when about to be examined, as necessary vouchers of their having attained the age of twenty-two; and I will venture to affirm, more than half the whole number of students that pass the hall, impose on the court of examiners in this manner. I remain, Sir,

Your obedient Servant,

C. W. S.

---

*To the Editor of the Medical and Physical Journal.*

